# Recurrent Oral Ulcers and Its Association With Stress Among Dental Students in the Northeast Indian Population: A Cross-Sectional Questionnaire-Based Survey

**DOI:** 10.7759/cureus.34947

**Published:** 2023-02-13

**Authors:** Saumya Verma, K Srikrishna, Srishti ., Kumari Shalini, Gunjan Sinha, Parul Srivastava

**Affiliations:** 1 Department of Oral Medicine and Radiology, Hazaribag College of Dental Sciences and Hospital, Hazaribagh, IND; 2 Department of Dentistry, Mahesh Ratna Eye and Dental Care and Research Center, Patna, IND

**Keywords:** questionnaire, survey, perceived stress scale, apthous ulcer, recurrent oral ulcer

## Abstract

Aim

The term “aphthous” is derived from the Greek word “aphtha,” which means ulceration. The cause of aphthous ulcers is multifactorial, but emotional stress is one of the most important risk factors for its occurrence in young individuals. The aim of this study was to evaluate the association of recurrent oral ulcers (ROUs) with stress among dental students in the Northeast Indian Population.

Materials and methods

A total of 1,134 students were recruited for the study. Two sets of questionnaires were designed. The first set consisted of 11 questions related to demographic data and information about recurrent oral ulcers. The second set of questionnaire had 14 questions related to stress symptoms. Google Forms (Google, Inc., Mountain View, CA, USA) was used for recording the responses of the study participants. Questionnaires were sent to the participants through e-mail, and responses were recorded. Statistical analysis was done using the Statistical Package for the Social Sciences (SPSS) version 21 (IBM SPSS Statistics, Armonk, NY, USA) and Microsoft Office Excel (Microsoft Corp., Redmond, WA, USA). The chi-square test was used to compare categorical variables.

Results

Among the 1,134 participants, 32.7% (371 students) reported a previous history of recurrent oral ulcers. Out of 371 participants who had given a history of oral ulcers, only 27.2% exhibited direct stress to ulcer association. On further assessment using the Perceived Stress Scale (PSS), it was found that a far greater percentage of students (i.e., more than 27.2%) were under some form of stress or the other.

Conclusions

The results of this study will help improve the quality of life among the study population, either by tackling their stress levels or by identifying adequate interventions.

## Introduction

Recurrent oral ulcers (ROUs) are one of the most common painful oral mucosal diseases. The causes of ROUs are multifactorial, but emotional stress is one of the most important risk factors for its occurrence in young individuals [1]. Stress is not only thought to be the cause of ROUs but also has an enormous impact on physical health and may lead to various systemic problems such as cardiovascular diseases and neurological disorders, among others. Various predisposing factors, such as stress, physical or chemical trauma, infection, allergy, genetic predisposition, or nutritional deficiencies, may contribute to ROUs. Among dental students, stress can be a significant threat and may exert a negative impact on physical and mental health, which will lead to poor academic performance and quality of life [2].

ROUs are quite frequent, affecting around 20% of otherwise normal people. This condition often manifests itself in the early stages of puberty and lasts for a few days before it heals completely. These ulcers tend to develop on a cyclical basis, and as they heal, they leave no scars. These ulcers typically persist between six and eight days and flare up between three and 12 times a year [3-5]. Various studies showed major areas of strength between the safe framework parts (distribution, multiplication, and movement of lymphocytes and regular executioner cells, phagocytosis, and creation of cytokines and antibodies) and the development of ROUs, which may explain the role of pressure in the event of ROUs [6].

Stress and anxiety have been shown to play a significant role in the emergence of ROUs since both states are associated with a transient elevation of salivary cortisol, which in turn stimulates immunologic regulatory function by elevating the quantity and activity of leukocytes [7]. A variety of hormonal and physiological responses occur in the body in response to being pushed. Concern sends signals to the brain, which in turn communicates with the main pituitary gland and the adrenal glands, resulting in the production of hormones such as norepinephrine, adrenaline, and cortisol, all of which have different effects on the body and may eventually lead to a cover-up of the host’s immunity. Multiple small, round, or oval ulcers with well-defined margins are the clinical hallmark of ROU. It manifests first during adolescence or early adulthood and is characterized by a yellowish or dark-white fibrinous effusion that is surrounded by an erythematous corona [8].

Patients’ quality of life is negatively impacted by ROUs because of the severe discomfort they experience during eating, deglutition, and speaking. However, the underlying cause of this ailment is still unclear; thus, most therapies focus on relieving symptoms and providing emotional support. The development of lesions may be precipitated by a number of different circumstances that include but are not limited to local trauma, hormonal changes, infectious agents including human immunodeficiency virus (HIV), vitamin inadequacy, drug consumption, predisposing factors, immune abnormalities, and family history. However, it has been shown that smoking reduces ROU prevalence through increasing mucosal keratinization.

Recurrent aphthous stomatitis (RAS) is a frequent ROU. The lips, the tongue’s ventral side, the buccal mucosa, the floor of the mouth, and the soft palate are common sites where RAS manifests. They are often painful, superficial round to oval ulcers with an erythematous base and covered by a yellowish-gray pseudomembrane, classified into Stanley’s three categories of RAS: minor RAS, major RAS, and herpetiform ulcers. Minor aphthous ulcers make up the bulk of RAS cases (80%). The non-keratinized mucosa is affected, and they typically recover in 10-14 days with little scarring and are only 8-10 mm in size and 1-5 in number. Large aphthous ulcers, which account for 10%-15% of RAS and often measure >1 cm in diameter, may spread to the keratinized oral mucosa and affect structures such as the hard palate. Scarring is common after healing, which typically takes around six weeks. Herpetiform RAS is characterized by clusters of tiny ulcers, often between 10 and 100 in number, with ulcer diameters of 1-3 mm. Most of the time, these ulcers heal without leaving any scars and only linger for around 10-14 days [9].

Numerous studies have demonstrated that stressful conditions can lead to the development of ROUs in dental students. Hence, to help students cope with these challenges, educators must raise their level of understanding on how to prevent ROUs. Although stress is something everyone experiences, the connection between stress and mouth ulcers is not well acknowledged. Therefore, the purpose of this research was to definitively link stress to the development of oral ulcers in dentistry students and argue for frequent psychological counseling of these individuals in an effort to alleviate their stress levels. This research intends to investigate the connection of recurrent oral ulcers with stress among dentistry students of the Northeast Indian population. This will make it simpler to treat and better manage mouth ulcers caused by stress.

## Materials and methods

This was a cross-sectional study conducted among 1,134 dental students from Northeastern India. A review was done at our institution’s Department of Oral Medicine and Radiology from February 2020 to March 2021. After obtaining ethical clearance, with ethics certificate number HCDSH/ADM/BNF/2020/213 dated 25/01/2020, each person who took part in the study after being selected by a census sample method gave their online informed consent. Only students who agreed to take part in the research were included; those with severe systemic diseases or who were taking drugs such as steroids, which suppress the immune system, were not included. Surveys were sent online (through Google Forms (Google, Inc., Mountain View, CA, USA) sent via e-mail and WhatsApp) to gather the necessary information. The questionnaires had two sets of questions. The first set consisted of 11 questions related to demographic data and clinically relevant information about recurrent oral ulcers (i.e., previous history of recurrent oral ulcers, family history of recurrent oral ulcers, time of last oral ulcer, duration of ulcer, frequency of ulcer, whether the ulcer was painful, number of ulcers noticed in each episode, area of occurrence, medication required, associated condition, and form of stress) (Table 1).

**Table 1:** First set of questionnaire

Demographic data and recurrent oral ulcer information
1. Previous history of recurrent oral ulcers
2. Family history of recurrent oral ulcer
3. Time of the latest ulcer
4. Duration of ulcer
5. Frequency of ulcer
6. Is the ulcer painful?
7. Number of ulcers noticed in each episode
8. Area of occurrence
9. Medication required
10. Association with any condition
11. Form of stress

The second set of questionnaires was composed of 14 questions that included questions related to stress symptoms such as the following: how frequently did anything unexpectedly happen that caused you discomfort in the last month; how frequently have you felt helpless during the last month with regard to life’s most fundamental aspects; if you were to rate how frequently you felt worried and “tense” throughout the last month, how often would you rate it as “very often”; how frequently in the previous month have you found a constructive solution to a bothersome problem; in the previous 30 days, what percentage of the time did you feel like you were effectively managing major life changes; how often in the previous month have you felt confident in your ability to tackle your personal difficulties; how frequently do you feel that things have been working out for you in the last month; how often in the previous month have you been overwhelmed by all you had to do; how frequently have you been able to stop things that were bothering you in the last month; how frequently have you felt in control this last month; how often have you been irritated by circumstances outside your control in the previous 30 days; how frequently have you been reminded in the last month that you have responsibilities that need to be fulfilled; how frequently have you been able to decide how you are going to spend your time in the previous month; and how often in the previous month have you felt that problems were building up to the point where you just could not get through them (Table 2)?

**Table 2:** Second set of questionnaire

Stress symptoms
1. In the past month, how often have you been upset because of something that happened unexpectedly?
2. In the past month, how often have you felt that you were unable to control the important things in your life?
3. In the past month, how often have you felt nervous and “stressed”?
4. In the past month, how often have you dealt successfully with irritating life hassles?
5. In the past month, how often have you felt that you were effectively coping with important changes that were occurring in your life?
6. In the past month, how often have you felt confident about your ability to handle your personal problems?
7. In the past month, how often have you felt that things were going your way?
8. In the past month, how often have you found that you could not cope with all the things that you had to do?
9. In the past last month, how often have you been able to control irritations in your life?
10. In the past month, how often have you felt that you were on top of things?
11. In the past month, how often have you been angered because of things that happened that were outside of your control?
12. In the past month, how often have you found yourself thinking about things that you have to accomplish?
13. In the past month, how often have you been able to control the way you spend your time?
14. In the past month, how often have you felt difficulties were piling up so high that you could not overcome them?

The respondents indicated their frequency of occurrence on a scale from 0 to 4 as follows: 0, never; 1, nearly never; 2, occasionally; 3, very often; and 4, very frequently. Psychological stress was assessed through a questionnaire, namely, the modified Perceived Stress Scale (PSS), suggested by Cohen et al. in 1983 [8]. The total stress score was calculated by adding the scores on 14 items, including four positively worded questions (questions 4, 5, 7, and 8) with responses that are reversed (0 = 4, 1 = 3, 2 = 2, 3 = 1, and 4 = 0) to the answers to the remaining 10 negatively worded questions (questions 1, 2, 3, 6, 9, 10, 11, 12, 13, and 14), with answers that are straightforward (0 = 0, 1 = 1, and so on). The overall stress scores obtained were compared for those individuals with or without recurrent oral ulcers. Further, a comparison of the obtained stress scores was done among all participants.

The Statistical Package for the Social Sciences (SPSS) version 21 (IBM SPSS Statistics, Armonk, NY, USA) and Microsoft Office Excel (Microsoft Corp., Redmond, WA, USA) were used for the statistical analysis. Statistical significance was determined at P values of less than 0.05 using the chi-square test used to compare categorical variables.

## Results

The present study was carried out among 1,134 dental students, out of which 32.7% (371 students) reported a previous history of recurrent oral ulcers. Among these 371 students, 5.7% (n=21) had ulcers at the time of the study, 20.8% (n=77) of them had ulcers one month back, 9.4% (n=35) had ulcers three months back, 11.3% (n= 42) had ulcers six months back, and 52.8% (n=196) of them had ulcers more than six months back. The majority (i.e., 58.5% (n=217)), out of 371, were having a single ulcer during each episode, whereas 41.5% (n=154) had 2-6 ulcers during each episode, and 0 were having more than six ulcers at each episode. Of them, 63.1% (n=234) had ulcers lasting for 3-6 days, 10% (n=37) had ulcers lasting for 7-10 days, and 27% (n=100) had ulcers lasting for 0-2 days. The predominant area of occurrence was the lips (35.8% (n=133)), followed by the cheek (34% (n=126)), gums (5.7% (n=21)), and tongue (3.8% (n=14)) (Table 3).

**Table 3:** Responses given by the participants

Questions	Responses
Previous history of recurrent oral ulcers	Yes (371 (32.7%)), no (763 (67.3%))
Family history of recurrent oral ulcers	Yes (336 (29.6%)), no (798 (70.4%))
Time of last oral ulcer	>6 months (196 (52.8%)), one month (77 (20.8%)), three months (35 (9.4%)), six months (42 (11.3%)), experiencing presently (21(5.7%))
Duration of ulcer	0-2 days (100 (27%)), 3-6 days (234 (63.1%)), 7-10 days (37 (10%))
Frequency of ulcer	Once in three months (79 (21.3%)), once in six months (239 (64.4%)), once in a month (30 (8.1%)), once in a week (23 (6.2%))
Is the ulcer painful?	Yes (270 (72.8%)), no (101 (27.2%))
Number of ulcers noticed in each episode	>6 (0 (0%)), 1 (217 (58.5%)), 2-6 (154 (41.5%))
Area of occurrence	Cheek (126 (34%)), gums (21 (5.7%)), lips (133 (35.8%)), multiple areas (77 (20.8%)), tongue (14 (3.8%))
Medication required	Doctor consultation/advice (14 (3.8%)), home remedy (77 (20.8%)), self-healing/not required (175 (47.2%)), topical gels (105 (28.3%))
Associated with any condition	Fever (31 (8.4%)), gastric problem (144 (38.8%)), hormonal change (54 (14.6%)), skin problem (7 (1.9%)), stress (101 (27.2%)), trauma (34 (9.2%))
Form of stress	Change in food (33 (8.9%)), examination (50 (13.5%)), loss of near and dear ones (16 (4.3%)), multiple reasons (155 (41.8%)), others (117 (31.5%))

**Figure 1:**
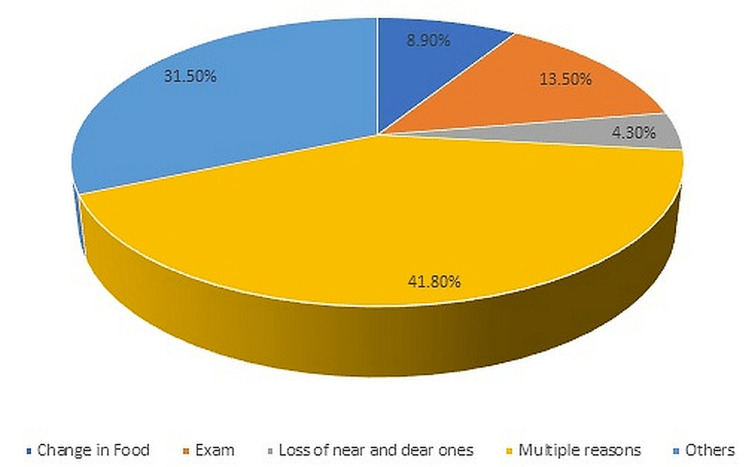
Frequency of stress distribution due to various causes

Among 371 students, 21.3% (n=79) experienced an ulcer once in three months, 64.4% (n=239) experienced an ulcer once in six months, 8.1% (n=30) experienced an ulcer once in a month, and 6.2% (n=23) experienced ulcer once in a week. The majority of the participants (i.e., 72.8% (n=270)) were experiencing painful oral ulcers, whereas 27.2% (n=101) had not experienced pain. The vast majority of those who took part in the study did not use any prescription drugs (i.e., 47.2% (n=175)), whereas 28.3% (n=105) had applied topical gels and 20.8% (n=77) had used home remedies, and only a few required doctor consultation (i.e., 3.8% (n=14)) (Table 3). About 29.6% (n=336) of the people in the research said that they had a positive family history, which is a significant number (p=0.004). With respect to the 371 contributors, a majority had gastric issues (38.8% (n=144)), whereas 27.2% (n=101) reported stress as the cause of ulcers, 14.6% (n=54) reported that their ulcerations were related to hormonal change, 9.2% (n=34) said the ulcers were related to trauma, 8.4% (n=31) reported the ulcers were associated with fever, and only 1.9% (n=7) reported that their ulcerations were related to skin problem (Figure 1).

Among 101 students who reported stress as the cause of ulcers, 13.5% reported examination as the form of stress, 8.9% reported change in food, and 41.5% had multiple reasons. On further assessment with the Perceived Stress Scale (PSS), it was found that students who had not revealed any history of ROU (n=763) were under more stress when compared with the group who had ulcers. Among these students (n=763), the PSS scores were high among 306 students (40.17%), 163 students (21.3%) had stress in the average range, and 294 students (38.4%) were under low stress. Stress among the students with ulcers was compared using the chi-square test, and it was found to be significant (Table 4).

**Table 4:** Comparison of stress among patients with ulcers and without ulcers (percentage) Q: question, *: significant

	History of ulcer	0 (%)	1 (%)	2 (%)	3 (%)	4 (%)	P value
Q1	Yes=371	16.98	9.43	43.40	5.66	24.53	0.001*
	No=763	30.67	15.20	29.36	15.60	9.17	
Q2	Yes=371	17.25	7.01	32.35	20.75	22.64	0.001*
	No=763	29.88	23.20	24.90	13.76	8.26	
Q3	Yes=371	5.93	8.89	21.02	26.42	37.74	0.001*
	No=763	23.07	9.83	28.57	19.27	19.27	
Q4	Yes=371	5.93	12.67	41.78	18.87	20.75	0.001*
	No=763	24.90	13.63	33.94	20.18	7.34	
Q5	Yes=371	15.36	14.56	39.89	16.98	13.21	0.001*
	No=763	27.00	16.91	34.86	17.56	3.67	
Q6	Yes=371	9.70	14.56	38.01	18.87	18.87	0.001*
	No=763	22.02	10.88	25.56	27.79	13.76	
Q7	Yes=371	9.43	33.42	40.16	11.32	5.66	0.001*
	No=763	33.55	19.53	36.44	8.52	1.97	
Q8	Yes=371	11.32	20.22	45.55	15.09	7.82	0.001*
	No=763	33.29	16.25	29.10	18.61	2.75	
Q9	Yes=371	9.43	16.44	38.27	20.75	15.09	0.001*
	No=763	26.21	12.45	30.93	21.23	9.17	
Q10	Yes=371	18.87	29.65	42.05	3.77	5.66	0.001*
	No=763	33.81	22.67	33.16	7.60	2.75	
Q11	Yes=371	11.32	12.94	34.23	30.19	11.32	0.001*
	No=763	23.85	11.66	32.11	18.35	14.02	
Q12	Yes=371	9.70	10.24	31.00	30.19	18.87	0.001*
	No=763	31.19	15.73	30.67	12.32	10.09	
Q13	Yes=371	6.20	26.68	42.59	13.21	11.32	0.001*
	No=763	25.82	10.22	42.33	12.19	9.44	
Q14	Yes=371	14.02	20.75	31.54	22.64	11.32	0.001*
	No=763	32.11	15.86	30.54	15.07	6.42	

## Discussion

This research sets out to determine whether dental undergraduates in a Northeast Indian population were more likely to have recurrent oral ulcers as a result of stress. It has been shown in the literature that ROUs may be triggered by psychological stress, which is often seen at times of high stress, such as final examinations, dental work, or major life transitions. Although the precise function of stress remains unclear, it has been linked to changes in the number, location, and function of immune system cells such as lymphocytes and natural killer T cells, as well as the release of cytokines and antibodies. Alterations to the immune system have also been related to ROUs, which may account for the hypothesis that stress plays a part in their development. Other possible causes of mouth ulcers include an increase in salivary cortisol or reactive oxygen species. Para-functional oral behaviors, such as lip and cheek biting, have been linked to stress-related anxiety, and the resulting physical damage may trigger the ulcerative process in vulnerable people [10-12].

Since mental health is so vital and since psychiatric disorders often play a part in the onset of ROUs, it is possible that individuals with ROUs may need to undergo psychiatric counseling or psychological therapy as part of their treatment. Interactions between dietary, hematologic, and genetic variables have been documented to further complicate the etiopathogenesis of ROUs. There was a long-held belief that psychological strain was a contributing factor in the development of certain diseases. Although the exact processes by which stress leads to ROU episodes remain unknown, it has been hypothesized that increased salivary cortisol or reactive oxygen species in the saliva may be the germinal germs for the ulcers to germinate. However, investigations have shown that individuals with ROUs have normal levels of myeloperoxidase and reactive oxygen species. Because of the stressful condition, patients may injure their oral mucosa by biting their cheeks or lips, which leads to the development of oral ulcers. Alterations in the genetic pathways may also be linked to stressful reactions that occur within the body. Another research indicated that the polymorphism in the serotonin transporter gene, which is often identified in individuals with anxiety or stress, was considerably greater in the group of ROU patients compared to the general population [13-16].

Oral lesions caused by ROUs may be very painful and make it difficult to talk, eat, or drink. It is hypothesized that stress might set in motion the processes responsible for the onset of ROUs, resulting in pain that can have deleterious effects on the person and elicit even more stress. Unfortunately, this may cause a vicious cycle in which stress causes ulcers, which in turn causes more stress [17]. Researching the frequency of ROUs is crucial because it reveals both the prevalence of the problem and the variables that may be contributing to it among students. The percentage of kids who have ROUs and require dental care may be estimated by learning more about the disease’s incidence and geographic distribution in Northeast Indian communities. Oral doctors may benefit from knowing the higher prevalence of recurrent oral ulcers to make a correct diagnosis. It will also help in providing different treatment information to the patient that might improve their condition [18].

The pathophysiologic impact of mental stress is not uniform for every individual. Sometimes, it might have complex and multiple mechanisms. Even if two people are experiencing the same level of stress or worry, they may exhibit it in very different ways. The purpose of this research was to determine how common recurrent oral ulcers are among dentistry graduate students and whether or not they are linked to emotional distress. Multiple studies have shown that emotional or mental strain significantly increases the probability of ROUs. Therefore, in the absence of dietary and other recognized predisposing variables, measures to avoid and manage stress may be helpful in minimizing the recurrence of ROU [19].

ROUs afflict around 20% of the population overall; however, the incidence might vary from 5% to 50% when studying certain ethnic or socioeconomic groups. In some communities, such as those studying medicine and dentistry, various studies demonstrated a high frequency of 50%-60% [20]. In those predisposed to anxiety, stress has been linked to the development of ROUs. Kasi et al. in 2007 showed that medical students experience high amounts of stress, leading many to resort to unhealthy ways of dealing with their anxiety [21]. The occurrence of reduced secretion of saliva caused xerostomia (dryness of the mouth) and oligosialia, which were considered important predisposing factors for the development of recurrent oral ulcers [7].

Since mouth ulcers commonly impede speech and other activities generating psychological influence on the person, thus diminishing the quality of life, the impact of various oral ulcers on the quality of life has increasingly been acknowledged as an essential outcome measure for therapeutic studies. Changes in one’s life that require adjusting one’s coping mechanisms and social circles may cause stress, which in turn can throw off one’s internal balancing act and lead to maladaptive behavioral patterns and even medical problems. Psychological stress was revealed to be a major triggering element in the onset of recurring mouth ulcers, even when stress and anxiety levels were similar to those of normal persons [4,8]. The pathophysiological consequences of stress differ from individual to individual, in particular, the dynamic and complicated systems that have varying effects on various people. As a result, the same patient may exhibit varying degrees of symptomatology in response to the same amount of psychological stress [2].

In this study, out of 371 participants who had a history of oral ulcers, only 27.2% exhibited a direct stress-ulcer association. On further assessment with the PSS [8], it was found that a far greater percentage of students (i.e., more than 27.2%) were under some form of stress or the other. This data emphasizes that a significant number of students who could not recall or associate stress as a cause of their ulcer were in fact under some type of stress.

In our study, comparatively few (32.7%) of the patients experienced mouth ulcers to the 2016 research by George et al. According to his findings, 46.2% of students were stressed out, as measured by a high PSS score; this may be because the survey was done at examination time when students were likely more anxious than usual about upcoming tests and pressured to finish assigned work [9]. The results pertaining to the site of ulcers and the number of ulcers in our study were similar to that of the study conducted by Safadi in 2009, i.e., a maximum number of students (35.8%) reported ulcers on the lips and cheeks, and having a single ulcer during each episode [10]. The results pertaining to the requirement and use of medications were similar to that of George et al., i.e., the maximum number of participants (47.2%) did not require any medication [9].

A number of studies associate anxiety, depression, and psychological stress with recurrent oral ulcers, but it is also prudent here to mention that in a study conducted by Pedersen in 1989, no association was found between stress and recurrent oral ulcers; the differences can be attributed to the change in the curriculum and the increase in competitiveness among the students [11]. In this study, the main predisposing factor for recurrent oral ulcers was found to be gastrointestinal tract (GIT) issues (38.8%), followed by stress (27.2%). Other factors that were also associated were hormonal change, trauma, and fever. A similar study by Rathod et al. in 2017 among 500 students and Al-Johani in 2019 among 245 dental students found that stress is the main predisposing factor for recurrent oral ulcers in the studied population; this can be attributed to the different dietary patterns. The diet in the East Indian region of Jharkhand tends to be spicy, and the eating habits tend to be irregular in the study subpopulation [12,13].

Multiple research has shown a link between recurrent oral ulcers, and emotional and mental stressors such as anxiety and sadness. However, a 1989 study conducted by Pedersen on 22 patients revealed no link between stress and recurrent oral ulcers, leading the researchers to conclude that further controlled studies are required to prove the existence of any link between stress and increased keratinization of the oral mucosa [11]. This study also revealed a positive family history, and George et al. also showed a strong link between hereditary factors and recurrent oral ulcers among 106 medical students in 2016 [9], as well as Safi et al. among 245 dental students in 2021 [14]. Hence, acute psychological stress influences the development of recurrent oral ulcers. Thus, stress management strategies such as relaxation may be offered in lowering ROU’s symptoms as well as reoccurrence.

Clinical relevance

This study’s findings will aid in the evaluation of dental students’ psychological stress and mental health, and they will also shed light on the connection between psychological factors and recurrent oral ulcers. The findings of this research contribute to bettering the lives of the people under investigation, either by tackling their stress levels or by identifying adequate interventions.

Limitations and future prospects

The study is limited by the small sample size and the inflexibility of closed-ended questions. We recommend further studies incorporating a larger sample size and open-ended questions for the designed questionnaire.

## Conclusions

Although dental students generally did not report feeling stressed, we discovered a substantial correlation between stress and recurrent oral ulcers when using the Perceived Stress Scale to assess mental health. As the stress level was very high among dental students, periodic psychological counseling sessions are required to reduce stress and improve the quality of life of the study subpopulation.
